# Complete chloroplast genome of the rare medicinal vegetable *Allium hookeri*

**DOI:** 10.1080/23802359.2021.2003262

**Published:** 2021-12-09

**Authors:** Fengming Ren, Liqiang Wang, Wei Zhuo, Dongliang Chen, Hongyan Huang, Lansheng Zhang

**Affiliations:** a College of Applied Technology, Lijiang Teachers College, Lijiang, China; b Chongqing Institute of Medicinal Plant Cultivation, Research and Utilization on Characteristic Biological Resources of Sichuan and Chongqing Co-construction Lab, Chongqing, China; c College of Pharmacy, Heze University., Heze, China; d Chongqing Shangyao Huiyuan Pharmaceutical co. LTD, Chongqing, China

**Keywords:** Chloroplast genome, *Allium hookeri*, medicinal vegetable, phylogenetic analysis

## Abstract

*Allium hookeri* is a rare medicinal plant with unique flavor. In this study, the first complete chloroplast (cp) genome of *A. hookeri* was sequenced and assembled based on the next generation sequencing. The cp genome is 153,592 bp in length, including a large single-copy (LSC) region of 82,609 bp, a small single-copy (SSC) region of 17,487 bp, and a pair of inverted repeat (IR) regions of 26,748 bp each. The genome encodes 131 genes, including 86 protein-coding genes, 39 tRNA genes, and six rRNA genes. The GC content of whole genome is 36.99%. The phylogenetic analysis based on 24 complete cp sequences revealed that *A. hookeri* was at the base of the phylogenetic tree, indicating an older species in the *Allium* genus.

*Allium hookeri* Thwaites is a member of the genus *Allium* in Amaryllidaceae. It is a rare plant, narrowly distributed in the 1500–4200 m mountainous areas of Southwest China, Sri Lanka, and Northern India (Xu and Rudolf 2000). *A. hookeri* exhibits high nutritional and medicinal values (Yang et al. 2017). It is rich in organosulfur compounds, polyphenols, and allicin (Li et al. 2014; Kima et al. 2016), which possesses biological activity such as anti-obesity (Park et al. 2018; Kim et al. 2019), anti-inflammatory (Kim et al. 2019; Lee et al. 2020), and antimicrobial (Li et al. 2014; Kima et al. 2016). Consequently, *A. hookeri* is not only used as a vegetable with unique flavor, but also it has been consumed as medicinal plant. However, only the chloroplast (cp) genome of *Allium ferganicum* was reported in the genus *Allium* (Liu et al., 2020) . There is no genomic information of *A. hookeri* that has been reported so far. In this study, the cp genome was sequenced for protecting and excavating the resource of *A. hookeri*.

Fresh leaves of *A. hookeri* were collected from Jinfo Mountain, Chongqing, China (107°24′ E, 29°17′ N, 1664 m). The voucher specimen was conserved in Chongqing Institute of Medicinal Plant Cultivation under the accession number of CIMPC-RFM-20210501 (contact person: Fengming Ren, pengyou@126.com). A modified CTAB-based method was used to extract the genomic DNA, and the purity and integrity of the DNA were analyzed by Nanodrop and agarose gel electrophoresis. The genomic DNA was used to generate libraries with insert size of 350 bp and generated about 14 Gb raw reads by Illumina Hiseq 2500 Platform (Illumina, Hayward, CA). The raw data from the platform was removed low-quality reads and adapters by trimmomatic (Bolger et al. 2014). Using the clean data with 150 bp paired-end read lengths obtained from the raw data, a cp genome was assembled by NOVOPlasty (Nicolas et al. 2016) and annotated by CPGAVAS2 (Shi et al. 2019). After manual check and adjustment, the annotated cp genome was submitted to GenBank (MZ557488).

The complete cp genome of *A. hookeri* was 153,592 bp long and exhibited a typical angiosperm circular cp structure, containing four regions: large single-copy region (LSC: 82,609 bp), small single-copy region (SSC: 17,487 bp), and a pair of inverted repeats (IRs: 26,748 bp). The GC content was 36.99% (whole genome), 34.83% (LSC), 30.01% (SSC), and 42.60% (IR). The GC content of the genome and each genomic region was also typical of angiosperm cp structure. The genome encoded 131 genes, including 86 protein-coding genes, 39 tRNA genes, and six rRNA genes.

A total of 23 whole genome sequences from the *Allium* of Amaryllidaceae were downloaded from the GenBank database. The genome sequence of *Dioscorea polystachya* was used as an outgroup. Finally, the 24 cp genome sequences were multi-aligned by MAFFT software (Katoh and Standley 2013). Based on the aligned sequences, a maximum-likelihood phylogenetic tree was built with 1000 bootstrap replicates by IQ-TREE (Nguyen et al., 2015)) under parameters of ‘-nt AUTO -m MFP -bb 1000 -bnni’. Phylogenetic analysis showed that *A. hookeri* was at the base of the phylogenetic tree, which was the oldest species in the selected *Allium* species (Figure 1).

**Figure 1.**
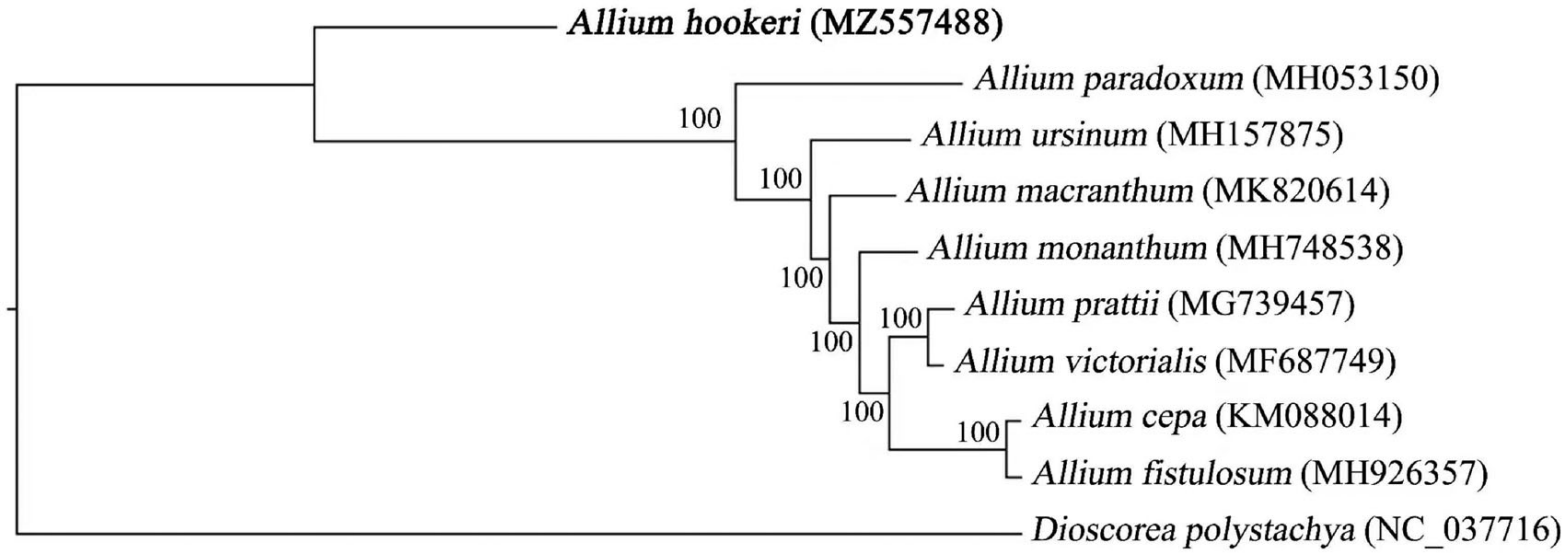
Maximum-likelihood phylogenetic tree based on the chloroplast genome sequences of eight *Allium* (Amaryllidaceae) species and *Dioscorea polystachya* (outgroup). The GenBank accession numbers is behind the Latin name. The bootstrap support values are beyond each node in the tree. *A. hookeri* is marked by bold font.

## Data Availability

The genome sequence data that support the findings of this study are openly available in GenBank of NCBI at https://www.ncbi.nlm.nih.gov/ under the accession no. MZ557488. The associated BioProject, Bio-Sample, and SRA numbers are PRJNA543381, SAMN20166845, and SRR15098630, respectively.
